# Pyronaridine-artesunate granules versus artemether-lumefantrine crushed tablets in children with *Plasmodium falciparum* malaria: a randomized controlled trial

**DOI:** 10.1186/1475-2875-11-364

**Published:** 2012-10-31

**Authors:** Kassoum Kayentao, Ogobara K Doumbo, Louis K Pénali, André T Offianan, Kirana M Bhatt, Joshua Kimani, Antoinette K Tshefu, Jack HT Kokolomami, Michael Ramharter, Pablo Martinez de Salazar, Alfred B Tiono, Alphonse Ouédraogo, Maria Dorina G Bustos, Frederick Quicho, Isabelle Borghini-Fuhrer, Stephan Duparc, Chang-Sik Shin, Lawrence Fleckenstein

**Affiliations:** 1 Malaria Research and Training Center, Faculté de Médecine de Pharmacie et d’Odonto-Stomatologie, Bamako, Mali; 2 Malariology Department, Institut Pasteur, Abidjan, Côte d'Ivoire; 3 UNITID, College of Health Sciences, University of Nairobi, Nairobi, Kenya; 4 Ecole de Santé Publique, Faculté de Médecine, Université de Kinshasa, Kinshasa, Democratic Republic of Congo; 5 Medical Research Unit, Albert Schweitzer Hospital, Lambaréné, Gabon; 6 Institute of Tropical Medicine, Tübingen, Germany; 7 Department of Medicine I, Division of Infectious Diseases and Tropical Medicine, Medical University of Vienna, Vienna, Austria; 8 Center National de Recherche et de Formation sur le Paludisme, Ministère de la Santé, Ouagadougou, Burkina Faso; 9 Research Institute for Tropical Medicine, Department of Health, FICC, Alabang, Muntinlupa City, Metro Manila, Philippines; 10 Medicines for Malaria Venture, International Center Cointrin, Route de Pré-Bois 20, PO Box 1826, CH-1215, Geneva 15, Switzerland; 11 Shin Poong Pharmaceutical, Seoul, South Korea; 12 University of Iowa, Iowa City, IA, USA

**Keywords:** Pyronaridine-artesunate, Artemether-lumefantrine, Malaria, *Plasmodium falciparum*, Pediatric

## Abstract

**Background:**

Children are most vulnerable to malaria. A pyronaridine-artesunate pediatric granule formulation is being developed for the treatment of uncomplicated *Plasmodium falciparum* malaria.

**Methods:**

This phase III, multi-center, comparative, open-label, parallel-group, controlled clinical trial included patients aged ≤12 years, bodyweight ≥5 to <25 kg, with a reported history of fever at inclusion or in the previous 24 h and microscopically-confirmed uncomplicated *P. falciparum* malaria. Patients were randomized (2:1) to pyronaridine-artesunate granules (60/20 mg) once daily or artemether-lumefantrine crushed tablets (20/120 mg) twice daily, both dosed by bodyweight, orally (liquid suspension) for three days.

**Results:**

Of 535 patients randomized, 355 received pyronaridine-artesunate and 180 received artemether-lumefantrine. Day-28 adequate clinical and parasitological response (ACPR), corrected for re-infection using polymerase chain reaction (PCR) genotyping (per-protocol population) was 97.1% (329/339; 95% CI 94.6, 98.6) for pyronaridine-artesunate; 98.8% (165/167; 95% CI 95.7, 99.9) for artemether-lumefantrine. The primary endpoint was achieved: pyronaridine-artesunate PCR-corrected day-28 ACPR was statistically significantly >90% (*P* < .0001). Pyronaridine-artesunate was non-inferior to artemether-lumefantrine: treatment difference -1.8% (95% CI -4.3 to 1.6). The incidence of drug-related adverse events was 37.2% (132/355) with pyronaridine-artesunate, 44.4% (80/180) with artemether-lumefantrine. Clinical biochemistry results showed similar mean changes versus baseline in the two treatment groups. From day 3 until study completion, one patient in each treatment group had peak alanine aminotransferase (ALT) >3 times the upper limit of normal (ULN) and peak total bilirubin >2xULN (i.e. within the Hy’s law definition).

**Conclusions:**

The pyronaridine-artesunate pediatric granule formulation was efficacious and was non-inferior to artemether-lumefantrine. The adverse event profile was similar for the two comparators. Pyronaridine-artesunate should be considered for inclusion in paediatric malaria treatment programmes.

**Trial registration:**

ClinicalTrials.gov: identifier NCT00541385

## Background

*Plasmodium falciparum* malaria kills approximately 850,000 children annually, most are under five years old [1,2]. The World Health Organization (WHO) generally recommends artemisinin-containing combination therapy (ACT) for the treatment of *P. falciparum* malaria [3]. However, of those medicines included on the WHO list of prequalified medicinal products, only artemether-lumefantrine dispersible tablets and artesunate-amodiaquine water-soluble tablets are available for oral liquid administration to children [4]. Clearly, there is a need for new formulations of ACT specifically formulated for pediatric use.

Pyronaridine-artesunate (3:1 ratio) is an ACT being developed for the treatment of uncomplicated *P. falciparum* and *Plasmodium vivax* malaria [5-7]. In two Phase III trials of pyronaridine-artesunate tablets, both conducted in adults and children in Africa and Asia with *P. falciparum* malaria, day-28 adequate clinical and parasitological response (ACPR) rates in the per-protocol population were 99.5% (780/784) and 99.2% (743/749) when corrected for re-infection with polymerase chain reaction genotyping (PCR-corrected) [5,6]. Pyronaridine-artesunate was non-inferior to artemether-lumefantrine and mefloquine plus artesunate, respectively [5,6]. The adverse event profile of pyronaridine-artesunate in these trials has been generally favorable, though liver transaminases were increased in some patients [5,6].

Children are most vulnerable to malaria [1,2,8-10] and adverse events are potentially more serious in this patient population. A pyronaridine-artesunate pediatric granule formulation was included in the development programme. A Phase II study in children described similar pharmacokinetics between the tablet and granule formulations [11]. This paper reports outcomes from a Phase III comparative, open-label, randomized, multi-center clinical study assessing the safety and efficacy of a fixed-dose oral pyronaridine-artesunate granule formulation (60:20 mg) versus artemether-lumefantrine crushed tablets in infants and children with acute uncomplicated *P. falciparum* malaria. The primary efficacy outcome of this trial was to demonstrate >90% efficacy of pyronaridine-artesunate granules in children with *P. falciparum* mono-infection evaluated using PCR-corrected day-28 ACPR in the per-protocol population [12]. A secondary efficacy outcome was to compare the efficacy of pyronaridine-artesunate granules with that of artemether-lumefantrine crushed tablets.

## Methods

### Ethics statement

The protocol was approved by each study center’s independent ethics committee and the study was conducted in accordance with the Declaration of Helsinki (Tokyo 2004), Good Clinical Practice, and applicable regulations. Informed written or witnessed oral consent was obtained from all patients’ parents/guardians; assent was required from children able to understand the study.

### Study design

This multi-center, comparative, randomized, open-label, parallel-group clinical trial followed WHO guidelines [12]. Study drugs were pyronaridine-artesunate granules (60/20 mg) supplied in aluminium sachets (Shin Poong Pharmaceutical Company, Ltd., Ansan, Korea), and artemether-lumefantrine tablets (20/120 mg), supplied in blister packs (Novartis SA, Basel, Switzerland). Patients were recruited from local hospitals and clinics at seven centers: Koupèla, Burkina Faso; Kinshasa, Democratic Republic of Congo; Lambaréné, Gabon; Anonkoua-koute, Côte d’Ivoire; Siaya, Kenya; Bougoula, Mali; and Puerto Princesa, the Philippines.

### Patients

Eligible subjects were of either sex, ≤12 years old, with a bodyweight ≥5 kg and <25 kg (with no evidence of severe malnutrition), a reported history of fever at inclusion or within the previous 24 h, and microscopically-confirmed uncomplicated *P. falciparum* mono-infection (asexual parasite density 1,000–200,000 μL^–1^ blood). If applicable, a negative pregnancy test was required. Subjects were excluded if they had: signs and symptoms of severe/complicated malaria [13]; mixed *Plasmodium* infection; severe vomiting (>3 times in the previous 24 h); severe diarrhoea (≥3 watery stools per day); other clinically significant disorders, including hepatitis or HIV infection; other febrile conditions; hepatic/renal impairment; electrolyte imbalance; anemia (hemoglobin <8 g/dL); hypersensitivity/allergy to study drugs; anti-malarial therapy in the previous two weeks, an investigational drug within four weeks, or any drug metabolized by cytochrome enzyme CYP2D6; participated previously in pyronaridine-artesunate clinical studies.

### Randomization and blinding

The sponsor provided a computer-generated randomization schedule. Patients were randomized 2:1 to pyronaridine-artesunate or artemether-lumefantrine. Randomization numbers were assigned in ascending order. Individually numbered treatment packs of similar appearance were masked on allocation. Clinical assessments and drug administration were performed by different clinical personnel. Drugs were given open-label. The study sponsor remained blinded to treatment allocation.

### Treatments

Study drugs were given orally for 3 days (days 0, 1 and 2), dosed according to bodyweight. All doses were directly observed. Pyronaridine-artesunate was given once daily: ≥5 to <9 kg, one sachet; 9 to <17 kg, two sachets; 17 to <25 kg, three sachets (dose range 6.7/2.2 to 13.3/4.4 mg/kg/dose). Artemether-lumefantrine was given twice daily: ≥5 to <15 kg, one tablet; 15 to <25 kg, two tablets (dose range 1.3/8.0 to 4.0/24.0 mg/kg/dose); the second day-0 dose was 8 h after the first dose, the first day-1 dose was 24 h after the first day-0 dose, with all subsequent doses 12 h apart.

Oral suspensions were prepared immediately before dosing. Pyronaridine-artesunate granules were stirred into 50 mL of water, milk, or soup. Artemether-lumefantrine tablets were crushed to coarse particles, added to 50 mL of water and shaken to a uniform suspension. Residual drug was given by adding 100 mL of water to the dosing cup. Artemether-lumefantrine was given with food or milk as per local guidelines. Vomiting within 30 minutes following the first drug dose resulted in re-dosing. Vomiting after repeat dosing or any subsequent dose resulted in study withdrawal and treatment with rescue medication (as per local guidelines).

### Procedures

At screening, a medical history was taken and a physical examination performed. Eligible patients were hospitalized from day 0 to day 3, with follow-up at days 7, 14, 21, 28, 35, and 42. Temperature was taken at screening, every 8 h over ≥72 h following the first dose or until two normal readings between 7 and 25 h apart, then at each visit or as clinically indicated. All study sites were provided with equipment which was used solely for the rapid analysis of biochemical and hematological samples obtained from the subjects evaluated for inclusion in the trial or samples obtained during the trial. Venous blood samples were taken for clinical biochemistry and hematology at screening, days 3, 7, 28, and 42; urinalysis was performed at screening. Electrocardiographs (ECG) were done at screening and day 2, and if indicated at days 7, 14, and 28.

Parasitological assessments were conducted according to WHO guidelines [12]. Venous blood samples for asexual and gametocyte parasite counts were taken before each dose, every 8 h (±1 h) following first dose administration for ≥72 h or until parasite clearance (two consecutive negative readings 7 to 25 h apart), and at subsequent visits. Giemsa-stained thick blood films were examined independently by two microscopists with the arithmetic mean recorded. A thin blood smear for species identification was examined at screening and from day 7 for all parasitological blood samples.

Following parasite reappearance, recrudescence and re-infection were distinguished by PCR genotyping performed at a central laboratory (Swiss Tropical and Public Health Institute, Basel, Switzerland). Using *P. falciparum* genes *msp1*, *msp2*, and *glurp*, recrudescence was defined as at least one matching allelic band in all markers between baseline and post-day-7 samples [14,15]. There were no amendments to the original study protocol.

### Endpoints

The primary efficacy endpoint was pyronaridine-artesunate day-28 PCR-corrected ACPR >90%. The main secondary efficacy endpoint was non-inferiority of pyronaridine-artesunate to artemether-lumefantrine for day-28 PCR-corrected ACPR [12]. Treatment failures were classified as early treatment failure, late clinical failure, and late parasitological failure according to WHO criteria [12].

Other secondary efficacy outcomes were: day-28 crude (non-PCR corrected) ACPR; day-42 PCR-corrected and crude ACPR; parasite clearance time (time from first dose until aparasitemia, i.e. two consecutive negative readings taken between 7 and 25 h apart); fever clearance time (time from first dose to apyrexia, i.e. two consecutive normal readings taken between 7 and 25 h apart); and the proportion of patients with parasite clearance or fever clearance on days 1, 2, and 3. Exploratory efficacy outcomes were: gametocyte density and proportion of patients with gametocytes; and gametocyte clearance time (defined as for parasite clearance time).

Safety outcomes were: adverse events, categorized using MedDRA (version, 10.1); laboratory abnormalities graded using the Division of Microbiology and Infectious Diseases Toxicity Scale (February, 2003); and ECG abnormalities.

### Sample size

A 95% cure rate was assumed for both treatments. For the primary efficacy endpoint, 320 evaluable patients in the pyronaridine-artesunate group provided 91% power to reject the null hypothesis (i.e. day-28 cure rate ≤90%) using a 1-sided exact binomial test with a nominal significance level of 2.5%. For the main secondary efficacy endpoint, 480 evaluable patients randomized 2:1 provided >99% power to demonstrate non-inferiority of pyronaridine-artesunate versus artemether-lumefantrine with a non-inferiority limit of 10%. Allowing for a 10% drop out rate, target recruitment was 534 subjects (356 pyronaridine-artesunate, 178 artemether-lumefantrine).

### Statistical analysis

The intent-to-treat population included all randomized subjects who received any study medication and was the same as the safety population. The per-protocol population included patients that received a full course of study medication, had a known day-28 primary endpoint, and did not violate the protocol so as to impair evaluation of the primary endpoint.

The primary efficacy endpoint was evaluated in the per-protocol population using the exact binomial test (significance limit ≤ .025). The associated exact (Pearson–Clopper) 2-sided 95% confidence interval (CI) was presented. The analysis was repeated for the intent-to-treat population. The primary efficacy endpoint was summarized by center, age category (<1 year, 1– < 5 years, 5–12 years), gender, previous episode of malaria (yes/no), and actual pyronaridine-artesunate dose (7.2/2.4 to 8.5/2.8 mg/kg, >8.5/2.8 to 9.5/3.2 mg/kg, >9.5/3.2 to 11.0/3.7 mg/kg, and >11.0/3.7 to 13.8/4.6 mg/kg).

The main secondary efficacy endpoint was evaluated in the per-protocol population. Pyronaridine-artesunate was non-inferior to artemether-lumefantrine if the lower limit of the 2-sided 95% CI (Newcombe–Wilson score method without continuity correction) for the difference between treatments was not lower than −10%. If pyronaridine-artesunate was non-inferior, superiority was tested using 2-sided Chi-square test (significance limit < .05). No multiplicity testing adjustment was required. The analysis was repeated for the intent-to-treat population, day-28 crude ACPR and day-42 PCR-corrected and crude ACPR.

All other analyses were presented for the intent-to-treat population. A post-hoc Kaplan–Meier analysis of recrudescence rate and re-infection rate was conducted and treatments compared using the log-rank test. Patients who did not have the event (recrudescence or re-infection) reported were censored at the last available parasite assessment date. In addition, patients with major protocol deviations were censored at the time of the deviations if they did not have the event before that time.

Parasite, fever and gametocyte clearance times were summarized using Kaplan–Meier estimates and treatment groups compared using the log-rank test. Patients without parasite or fever clearance within 72 h after the first drug dose were censored at that time point. The proportion of subjects with parasite, fever, and gametocyte clearance on days 1, 2 and 3 was calculated using Kaplan–Meier estimates. Statistical analysis was performed using SAS Version 9.1.3.

## Results

### Patients

**Figure 1:**
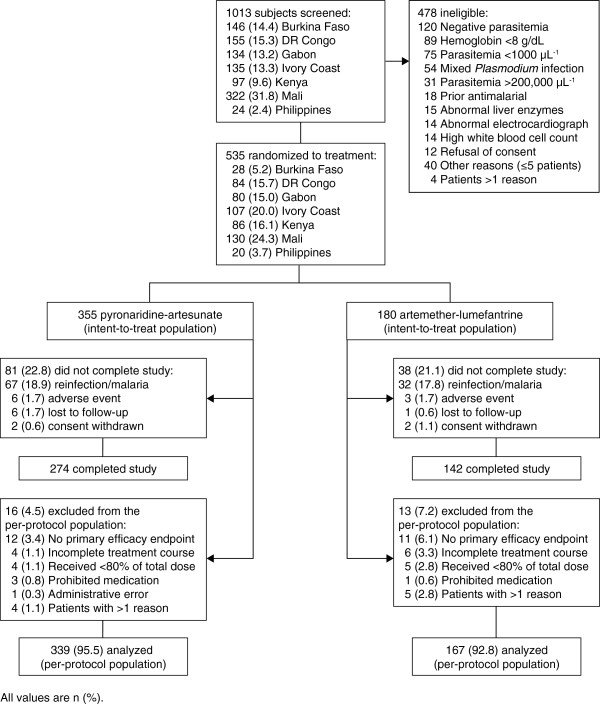
Study design and patient flow.

Between November 2007 and September 2008, 355 patients were randomized to pyronaridine-artesunate, 180 to artemether-lumefantrine (Figure 1). All randomized patients were included in the intent-to-treat and safety populations. Baseline demographic and clinical characteristics were similar between the two treatment groups (Table 1).

**Table 1:** Demographic and clinical characteristics of the intent-to-treat population at screening

**Characteristic**	**Pyronaridine-artesunate**	**Artemether-lumefantrine**
	***n*** **= 355**	***n*** **= 180**
Male, n (%)	177 (49.9)	84 (46.7)
Mean age, years (SD) [range]	4.9 (2.5) [0.3–11]	5.3 (2.5) [0.3–12]
<1 year, n (%)	12 (3.4)	3 (1.7)
1– < 5 years, n (%)	148 (41.7)	69 (38.3)
5–12 years, n (%)	195 (54.9)	108 (60.0)
Ethnicity, n (%)		
Black	342 (96.3)	172 (95.6)
Asian/Oriental	13 (3.7)	8 (4.4)
Mean weight, kg (SD) [range]	16.3 (4.1) [6.0–24.8]	17.1 (4.4) [7.2–24.9]
Mean body mass index, kg/m^2^ (SD) [range]	15.0 (2.2) [6.0–24.5]	15.1 (2.3) [10.7–25.0]
Geometric mean asexual parasites per μL	18565.9	18798.3
(95% CI)	(16005.9, 21535.4)	(15243.1, 23182.7)
Patients with gametocytes, n (%)	43 (12.1)	25 (13.9)
Temperature, °C	38.0 (1.1) [35.9–41.5]	37.8 (1.1) [35.4–41.0]
Fever at screening, n (%)	230 (65.0)	107 (59.4)
No previous malaria infection, n (%)	130 (36.7)	59 (32.8)
Malaria in last 12 months, n (%)^a^		
None	143 (41.1)	65 (36.7)
1	65 (18.7)	34 (19.2)
2	56 (16.1)	35 (19.8)
>2	84 (24.1)	43 (24.3)

^a^ Malaria in last 12 months; status was unknown for 10 patients.

### Efficacy

The primary efficacy endpoint was achieved. Pyronaridine-artesunate day-28 PCR-corrected ACPR in the per-protocol population was 97.1% (329/339; 95% CI 94.6, 98.6); statistically significantly >90% (*P* < .0001; Table 2). The intent-to-treat analysis was supportive of the primary analysis (*P* = .0077; Table 3).

**Table 2:** Adequate clinical and parasitological response (ACPR) in the per-protocol population

**Outcome**	**Pyronaridine-artesunate**	**Artemether-lumefantrine**	**Difference (95% CI);**
			***P *value**^**a**^
Day-28 PCR-corrected ACPR,^b^ n/N	329/339	165/167	
% (95% CI)	97.1 (94.6–98.6)	98.8 (95.7–99.9)	–1.8 (–4.3 to 1.6); *P* = .22
*P* value (exact binomial test)^c^	<.0001	NC	
Total failures	10 (2.9)	2 (1.2)	
Early treatment failure	2 (0.6)	0	
Late clinical failure	2 (0.6)	0	
Late parasitological failure	6 (1.8)	2 (1.2)	
Day-28 crude ACPR, n/N	305/341	149/172	
% (95% CI)	89.4 (85.7–92.5)	86.6 (80.6–91.3)	2.8 (–2.9 to 9.4); *P* = .35
Total failures	36 (10.6)	23 (13.4)	
Early treatment failure	2 (0.6)	0	
Late clinical failure	6 (1.8)	4 (2.3)	
Late parasitological failure	28 (8.2)	19 (11.0)	
Day-42 PCR-corrected ACPR,^b^ n/N	257/275	133/139	
% (95% CI)	93.5 (89.9–96.1)	95.7 (90.8–98.4)	–2.2 (–6.5 to 3.1); *P* = .36
Total failures	18 (6.5)	6 (4.3)	
Early treatment failure	2 (0.7)	0	
Late clinical failure	2 (0.7)	0	
Late parasitological failure	14 (5.1)	6 (4.3)	
Day-42 crude ACPR, n/N	249/325	130/166	
% (95% CI)	76.6 (71.6–81.1)	78.3 (71.3–84.3)	–1.7 (–9.1 to 6.4); *P* = .67
Total failures	76 (23.4)	36 (21.7)	
Early treatment failure	2 (0.6)	0	
Late clinical failure	14 (4.3)	5 (3.0)	
Late parasitological failure	60 (18.5)	31 (18.7)	

*NC*, not calculated. Values are n (%) unless otherwise indicated.

^a^ Non-inferiority of pyronaridine-artesunate to artemether-lumefantrine is concluded if the lower limit of the 95% CI for the difference is > –10%. Two-sided Chi-square test for superiority was performed only when non-inferiority was demonstrated.

^b^ Corrected for re-infection using polymerase chain reaction (PCR) genotyping.

^c^ For the hypothesis that the ACPR in the pyronaridine-artesunate group is ≤90%.

Note: There were no instances of indeterminate PCR results in the per-protocol population.

Per-protocol population: The per-protocol population was defined by time point (day 28 and day 42) and by endpoint (PCR-corrected ACPR versus crude ACPR). Patients with a new infection (re-infection) before day 28 were included in the day-28 per-protocol population for the crude analysis (failures). However, in the PCR-corrected analysis, patients with re-infection before day 28 were excluded owing to missing data at day 28, i.e. they were not deemed a failure (recrudescence) before day 28 and had missing data for this time point. The day-42 per-protocol population was defined similarly.

**Table 3:** Adequate clinical and parasitological response (ACPR) in the intent-to-treat population

**Outcome**	**Pyronaridine-artesunate**	**Artemether-lumefantrine**	**Difference (95% CI);**
			***P *value**^**a**^
Day-28 PCR-corrected ACPR,^b^ n/N	333/355	167/180	
% (95% CI)	93.8 (90.8–96.1)	92.8 (88.0–96.1)	1.0 (–3.2 to 6.2); *P* = .65
P value (exact binomial test)^c^	0.0077	NC	
Total failures	22 (6.2)	13 (7.2)	
Early treatment failure	2 (0.6)	0	
Late clinical failure	2 (0.6)	0	
Late parasitological failure	6 (1.7)	2 (1.1)	
Missing = failure	10 (2.8)	6 (3.3)	
Re-infection before day 28	2 (0.6)	5 (2.8)	
Day-28 crude ACPR, n/N	308/355	151/180	
% (95% CI)	86.8 (82.8–90.1)	83.9 (77.7–88.9)	2.9 (–3.2 to 9.7); *P* = .37
Total failures	47 (13.2)	29 (16.1)	
Early treatment failure	2 (0.6)	0	
Late clinical failure	6 (1.7)	4 (2.2)	
Late parasitological failure	29 (8.2)	19 (10.6)	
Missing = failure	10 (2.8)	6 (3.3)	
Day-42 PCR-corrected ACPR,^b^ n/N	271/355	140/180	
% (95% CI)	76.3 (71.6–80.7)	77.8 (71.0–83.6)	–1.4 (–8.6 to 6.4); *P* = .71
Total failures	84 (23.7)	40 (22.2)	
Early treatment failure	2 (0.6)	0	
Late clinical failure	2 (0.6)	0	
Late parasitological failure	15 (4.2)	6 (3.3)	
Missing = failure	12 (3.4)	6 (3.3)	
Re-infection before day 42	53 (14.9)	28 (15.6)	
Day-42 crude ACPR, n/N	264/355	136/180	
% (95% CI)	74.4 (69.5–78.8)	75.6 (68.6–81.6)	–1.2 (–8.6 to 6.8); *P* = .77
Total failures	91 (25.6)	44 (24.4)	
Early treatment failure	2 (0.6)	0	
Late clinical failure	14 (3.9)	5 (2.8)	
Late parasitological failure	63 (17.7)	33 (18.3)	
Missing = failure	12 (3.4)	6 (3.3)	

*NC*, not calculated. Values are n (%) unless otherwise indicated.

^a^ Non-inferiority of pyronaridine-artesunate to artemether-lumefantrine is concluded if the lower limit of the 95% CI for the difference is > –10%. Two-sided Chi-square test for superiority was performed only when non-inferiority was demonstrated.

^b^ Corrected for re-infection using polymerase chain reaction (PCR) genotyping.

^c^ For the hypothesis that the ACPR in the pyronaridine-artesunate group is ≤90%.

Note: There were no instances of indeterminate PCR results in the intent-to-treat population.

There were no statistical differences in the primary efficacy outcome observed by age, center, gender, or previous episode of malaria. However, there were some numerical differences by age and by dose that require further evaluation (see discussion). The efficacy of pyronaridine-artesunate for children aged <1 year was 81.8% (9/11; 95% CI 48.2–97.7), for children >1– < 5 years was 95.7% (135/141; 95% CI 91.0–98.4) and for children 5–12 years was 98.9% (185/187; 95% CI 96.2–99.9) (Additional file 1). The efficacy rate for a pyronaridine-artesunate dose of ≤8.5:2.8 mg/kg was 92.2% (107/116; 95% CI 85.8, 96.4); for >8.5/2.8 to 9.5/3.2 mg/kg, 100.0% (100/100; 95% CI 96.4, 100); for >9.5/3.2 to 11.0/3.7 mg/kg, 100.0% (97/97; 95% CI 96.3, 100); and for >11.0/3.7 mg/kg 96.2% (25/26; 95% CI 80.4, 99.9). Day-28 PCR-corrected ACPR for individual study centers was between 92.8% and 100% (Additional file 1).

Non-inferiority of pyronaridine-artesunate to artemether-lumefantrine was concluded for day-28 PCR-corrected ACPR in the per-protocol population (Table 2). Non-inferiority was also demonstrated for day-28 crude ACPR and day-42 PCR-corrected and crude ACPR in the per-protocol population (Table 2) and for outcomes in the intent-to-treat population (Table 3).

In the intent-to-treat population (Table 3), at day 28, data were available for 97.2% (345/355) of patients in the pyronaridine-artesunate group, with 10 cases of recrudescence and 27 cases of re-infection, i.e. 10.7% (37/345) of patients with data at this time point had parasite reappearance; for artemether-lumefantrine, data were available for 96.7% (174/180) of patients with 2 cases of recrudescence and 21 cases of re-infection, i.e. 13.2% (23/174) of patients with data at this time point had parasite reappearance.

At day 42 in the intent-to-treat population (Table 3), data were available for 96.6% (343/355) of patients in the pyronaridine-artesunate group, with 19 cases of recrudescence and 60 cases of re-infection, i.e. 23.0% (79/343) of patients with data at this time point had parasite reappearance; for artemether-lumefantrine, data were available for 96.7% (174/180) of patients with 6 cases of recrudescence and 32 cases of re-infection, i.e. 21.8% (38/174) of patients with data at this time point had parasite reappearance.

**Figure 2:**
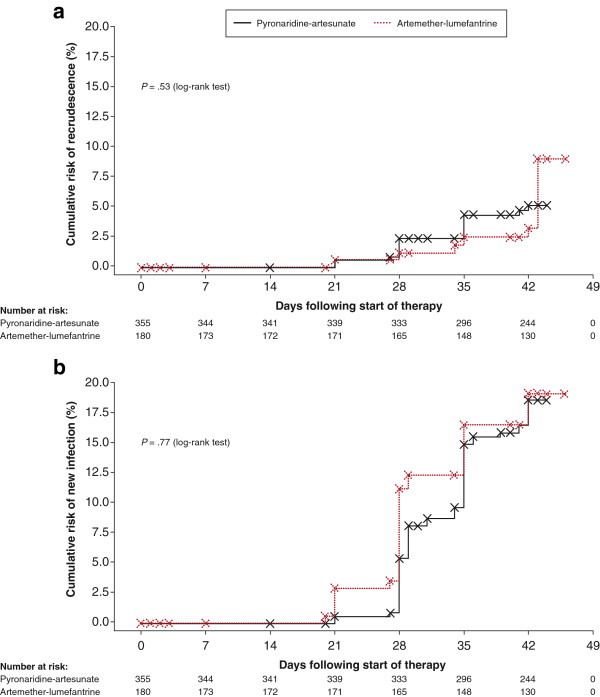
**Kaplan–Meier analysis (intent-to-treat population) showed no difference between pyronaridine-artesunate granules and artemether-lumefantrine crushed tablets for (a) recrudescence rate (*P*** **= .53, log-rank test) or (b) re-infection rate (*P*** **= .77, log-rank test).**

Kaplan–Meier analysis (intent-to-treat population) showed no difference between the two treatment groups for recrudescence rate (*P* = .53, log rank test; Figure 2a), or re-infection rate (*P* = .77, log rank test; Figure 2b).

**Figure 3:**
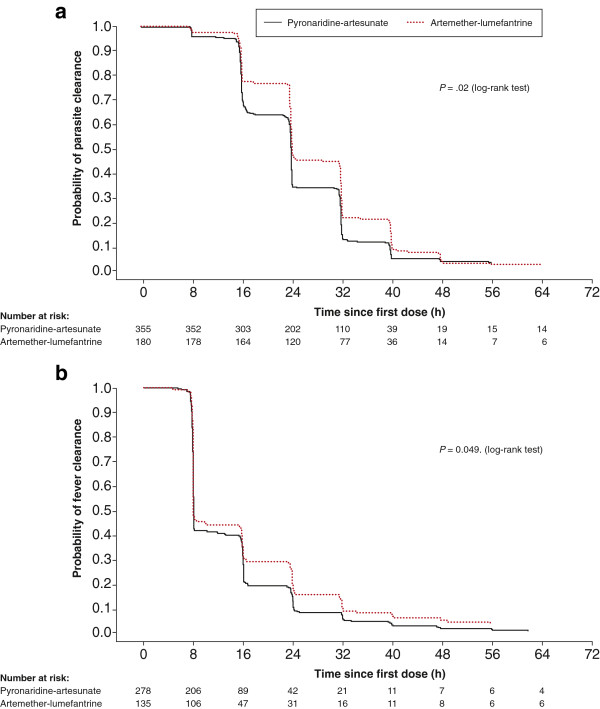
**Kaplan–Meier analysis (intent-to-treat population) showed significantly faster (a) parasite clearance time (*P*** **= .02, log-rank test) and (b) fever clearance time (*P*** **= .049, log-rank test) with pyronaridine-artesunate granules versus artemether-lumefantrine crushed tablets.**

Additional file 2 shows median asexual parasite, fever and gametocyte clearance times and the proportion of patients with clearance at days 1, 2, and 3. Median time to parasite clearance was shorter with pyronaridine-artesunate (24.1 h; 95% CI 24.0, 24.1) versus artemether-lumefantrine (24.2 h; 95% CI 24.1, 32.0; *P* = .02, log-rank test; Figure 3a). Anti-pyretic medication was taken by 220/355 (62.0%) patients in the pyronaridine-artesunate group and 118/180 (65.6%) in the artemether-lumefantrine group. Median fever clearance time was marginally shorter with pyronaridine-artesunate (8.1 h; 95% CI 8.0, 8.1) versus artemether-lumefantrine (8.1 h; 95% CI 8.0, 15.8; *P* = .049, log rank test; Figure 3b). At baseline, 13.1% (70/535) of patients had gametocytes. For those patients who had gametocyte clearance by day 3, median gametocyte clearance time was similar between the treatment groups (*P* = .48, log-rank test). Complete gametocyte clearance was achieved between day 41 and day 44 in the pyronaridine-artesunate group and between days 31 and 40 in the artemether-lumefantrine group. In the pyronaridine-artesunate group a total 26.8% (95/354) patients had post-baseline gametocytes versus 24.7% (44/178) with artemether-lumefantrine. New occurrences of gametocytes in patients that had none at baseline occurred in 15.0% (53/354) of patients in the pyronaridine-artesunate group and 11.2% (20/178) in the artemether-lumefantrine group.

During follow-up, there were five cases of *P. ovale* (three in the pyronaridine-artesunate group); all occurred on or after day 28. There were two cases of *P. malariae,* occurring at day 35 and day 42 (both in the artemether-lumefantrine group). There were no cases of *P. vivax* infection. All cases of non-falciparum malaria were treated as per local guidelines.

### Safety

Mean total drug exposure was 27.7/9.2 mg/kg (average daily dose 9.2/3.1 mg/kg) for pyronaridine-artesunate and 11.5/68.9 mg/kg (average daily dose 3.8/23.0 mg/kg) for artemether-lumefantrine.

Adverse events of any cause were experienced by 285/355 (80.3%) patients in the pyronaridine-artesunate, and 143/180 (79.4%) patients in the artemether-lumefantrine group (Table 4). Adverse events thought by the investigator to be drug related occurred in 132/355 (37.2%) patients in the pyronaridine-artesunate group and 80/180 (44.4%) in the artemether-lumefantrine group. There were no clinically important differences in the nature or incidence of adverse events between the two study groups (Table 4).

**Table 4:** Adverse events in the intent-to-treat (safety) population

**Outcome**	**Pyronaridine-artesunate**	**Artemether-lumefantrine**
	***n*** **= 355**	***n*** **= 180**
Adverse event of any cause^a^	285 (80.3)	143 (79.4)
Cough	44 (12.4)	28 (15.6)
Upper respiratory tract infection	42 (11.8)	19 (10.6)
Anemia	34 (9.6)	14 (7.8)
Platelets increased	33 (9.3)	19 (10.6)
Blood glucose decreased	32 (9.0)	19 (10.6)
Bronchitis	28 (7.9)	10 (5.6)
Vomiting	25 (7.0)	8 (4.4)
Pyrexia	23 (6.5)	8 (4.4)
Blood albumin decreased	21 (5.9)	16 (8.9)
Influenza-like illness	19 (5.4)	8 (4.4)
Drug-related adverse events^b^	132 (37.2)	80 (44.4)
Blood glucose decreased	29 (8.2)	15 (8.3)
Platelet count increased	27 (7.6)	14 (7.8)
Blood albumin decreased	19 (5.4)	16 (8.9)
Anemia	15 (4.2)	11 (6.1)
Blood potassium increased	15 (4.2)	6 (3.3)
Hemoglobin decreased	15 (4.2)	5 (2.8)
Upper respiratory tract infection	14 (3.9)	5 (2.8)
AST increased	14 (3.9)	7 (3.9)
Hematocrit decreased	14 (3.9)	5 (2.8)
Vomiting	7 (2.0)	6 (3.3)
Blood creatinine decreased	7 (2.0)	7 (3.9)

All values are n (%). AST, aspartate aminotransferase.

^a^ Experienced by ≥5% of patients in either treatment group.

^b^ Experienced by ≥3% of patients in either treatment group.

There were no deaths. One serious adverse event (complicated malaria) occurred in a 2-year-old male, resulting in study withdrawal on day 0 (11 h 20 mins after inclusion) after receiving one dose of pyronaridine-artesunate. He was treated with intramuscular artemether (day 1 and days 6–10) and intravenous quinine (days 1–5) and recovered well. The investigator noted this adverse event as unrelated to study treatment. In both treatment groups, 1.7% of patients had adverse events leading to drug discontinuation and study withdrawal: six patients receiving pyronaridine-artesunate (five with vomiting, one with malaria) and three receiving artemether-lumefantrine (three with vomiting).

Hematology results showed similar mean changes from baseline in the two treatment groups consistent with effective anti-malarial therapy. Mean hemoglobin concentrations decreased by –0.60 to –0.68 g/dL on day 3 and recovered by Day 28 (Additional file 3), with corresponding changes in hematocrit and red blood cell count. Mean increases in reticulocyte count of 0.5–0.6% were seen on Day 7 (Additional file 3). There were no other clinically relevant hematological changes.

Clinical biochemistry results showed similar mean changes versus baseline in the two treatment groups (Additional file 3). From day 3 until study completion, one patient in each treatment group triggered a potential Hy’s law case (i.e. peak alanine aminotransferase [ALT] >3 times the upper limit of normal [ULN] and peak total bilirubin >2xULN) [16]. In the patient receiving pyronaridine-artesunate, peak ALT was 704 U/L (day 7), though alkaline phosphatase (ALP) was elevated at screening (210 U/L), suggesting a possible underlying cause. In the patient receiving artemether-lumefantrine, peak ALT was 759 U/L (day 3), and alkaline phosphatase was normal (5 U/L) at screening. One additional patient in the artemether-lumefantrine group had peak AST >3xULN and peak total bilirubin >2xULN. In all cases, values were within normal limits at the final assessment (days 28–42). There were no clinically important changes in any other laboratory parameters.

There were no post-baseline clinically important abnormal ECG results. One patient in the artemether-lumefantrine group had an adverse event of arrhythmia, considered possibly drug related.

## Discussion

In children with uncomplicated *P. falciparum* malaria treated with pyronaridine-artesunate granules, day-28 PCR-corrected ACPR was 97.1% (95% CI 94.6, 98.6) in the per-protocol population. This is consistent with the high efficacy rates reported with pyronaridine-artesunate tablets in Phase III trials conducted in children and adults with *P. falciparum* malaria [5,6]. Introduction of a new anti-malarial requires an efficacy of >95% [3]. Statistically this trial was powered to show whether a cure rate of >90% was achieved. This was because of practical reasons in determining a reasonable sample size and the assumption that cure rates would be around 95%. Reaching an actual cure rate of 95% with a confidence interval of 95% ± Δ only allows you to demonstrate that the cure rate is >95% – Δ. Thus, with an actual cure rate of 95% it was deemed realistic to demonstrate that the cure rate is >90%. The sample size calculation was based on these assumptions.

Pyronaridine-artesunate was highly efficacious across all seven study centers and was equally effective across all age groups (Additional file 1). However, the primary efficacy outcome was not quite met in the youngest age group (81.8%; 95% CI 48.2–97.7), though only 11 children <1 year old were analyzed in the per-protocol population for pyronaridine-artesunate. In children aged 1–5 years, outcomes were similar to the older children and met the primary endpoint. Thus, further data are needed to assess to efficacy of pyronaridine-artesunate in very young children (<1 year old). Studies in very young children should only be conducted after more extensive clinical use in older children and adults to demonstrate safety. A study protocol could be envisaged where adults/older children are first recruited, and once a safety review of that population is complete, recruitment could be extended to a cohort of children more than two years of age and if safety is satisfactory in this group, finally, a cohort of very young children from 6 months old could be included in the study.

The data suggested an improved efficacy with pyronaridine-artesunate doses higher than 8.5:2.8 mg/kg/day, using PCR-corrected cure rates. These data are in contrast to a Phase II trial, in which PCR-corrected efficacy was 100% for pyronaridine-artesunate dose groups of 6:2 mg/kg, 9:3 mg/kg and 12:4 mg/kg [11]. Population pharmacokinetic data for pyronaridine indicate a larger volume of distribution in children versus adults, resulting in lower concentrations over time (author’s unpublished data). The limited immunity of children under 5 years of age to *P. falciparum* could also make this population more sensitive to dose–response effects. These findings will be examined more closely in a report on the population pharmacokinetics across the Phase III trials.

Pyronaridine-artesunate efficacy in children was non-inferior to that of artemether-lumefantrine; consistent with previous findings in children and adults [5]. There were no differences between treatment groups in the rate of recrudescence or re-infection (Figure 2). In the previous Phase III study (tablet formulation), pyronaridine-artesunate showed a significantly greater post-treatment prophylactic effect versus artemether-lumefantrine at day 42 (*P* = .007) [5]. This might result from differences in transmission rates between the two studies, or because in the previous study 56.7% of patients were >12 years old with no children under 5 years. Their greater immunity to *P. falciparum* may have contributed to a more sustained prophylactic effect.

As expected, both treatments reduced parasitemia rapidly. Parasite clearance time was shorter with pyronaridine-artesunate versus artemether-lumefantrine (*P* = .02). This was seen previously [5], and in studies of other forms of ACT containing dihydroartemisinin or artesunate compared with artemether [17,18]. Artesunate is converted more rapidly and completely than artemether to the active form dihydroartemisinin and has greater oral availability on the first day of treatment [19].

The effect of fat for optimizing artemether-lumefantrine absorption is well known and in this study we allowed each center to follow their local recommendations with regard to food or milk at the point of administration. Although this potentially introduces some variability, artemether-lumefantrine efficacy was high across all centers included in the study. There is no significant food effect with pyronaridine-artesunate which can be given regardless of food intake.

Both study treatments were generally well tolerated. Adverse events with pyronaridine-artesunate were consistent with those observed for both components given as monotherapy [20-23], and with previous clinical trials of the fixed-dose combination [5-7,11]. There were no clinically relevant differences in adverse events according to drug treatment or age group.

The incidence of peak ALT >3xULN plus peak total bilirubin >2xULN was 0.3% (1/355) in the pyronaridine-artesunate group and 0.6% (1/180) in the artemether-lumefantrine group. ALT elevations with increased bilirubin have been observed with pyronaridine-artesunate tablets at a similar incidence in two Phase III studies in *P. falciparum* in 5/1925 (0.3%) patients [5,6]. All cases were in adolescents or adults (14, 23, 25, 39 and 43 years old) and there were no clinical symptoms or evidence of liver injury [5,6]. There were no such cases in a trial of pyronaridine-artesunate in *P. vivax*[7].

Meta-analysis has suggested that pediatric ACT formulations have lower rates of drug-related gastrointestinal adverse events versus tablets [24]. Drug-related gastrointestinal adverse events were more common in a previous trial of adults and children receiving pyronaridine-artesunate tablets (vomiting 3.3%, other gastrointestinal 6.6%) versus artemether-lumefantrine (1.9%, 5.2%, respectively) [5]. This trend was reversed in the current study with pyronaridine-artesunate pediatric granules (vomiting 2.0%, other gastrointestinal 2.0%) versus artemether-lumefantrine crushed tablets (3.3% and 3.9%, respectively).

Pyronaridine-artesunate pediatric granules were efficacious and well tolerated in this study of children under 12 years of age with uncomplicated *P. falciparum* malaria. Considering these data and those of the other Phase III trials [5,6], pyronaridine-artesunate appears to be a valuable new ACT for use in both adults and children with *P. falciparum* malaria.

## Competing interest

IB-F and SD are employees of the Medicines for Malaria Venture, C-SS is an employee of Shin Poong Pharmaceutical Co. Ltd. There was no conflict of interest for KK, OKD, LKP, ATO, KMB, JK, AKT, JKHT, MR, PMdeS, ABT, AO, MDGB, FQ, or LF.

## Authors’ contribution

IB-F, SD, C-SS and LF made substantial contributions to the concept and design of the study. KK, OKD, LKP, ATO, KMB, JK, AKT, JKHT, MR, PMdeS, ABT, AO, MDGB, FQ and LF were involved in the acquisition of data. All authors contributed to the analysis and interpretation of data. All authors critically reviewed the paper and read and approved the final manuscript.

In addition to the named authors, the following co-investigators contributed to this study: Bakary Sidibé, Abdoulaye Djimdé (Mali); Berenger A. A. Ako, Aristide M'Lanhoro Coulibaly (Ivory Coast); Moses Omwoyo, Jacqueleen Wanjiru (Kenya); Nsengi Ntamabyaliro, Raoul Mpoyi Ngambua (Democratic Republic of Congo); Sabine Bélard, Florian Kurth (Gabon); Désiré Kargougou, David T. Kangoye (Burkina Faso); Jennifer Rabang (Philippines).

**Presentation:** This study was presented in part at the 5th MIM Pan-African Malaria Conference, Nairobi, Kenya 2–6 November, 2009. Kassoum Kayentao et al. Phase III pivotal trial of pyronaridine artesunate versus artemether lumefantrine in paediatric patients with acute uncomplicated *Plasmodium falciparum* malaria. Abstract MIM16689330.

## Supplementary Material

Additional file 1 PCR-corrected day-28 adequate clinical and parasitological response rates in the per-protocol population by country and patient age. Click here for file

Additional file 2 Number of patients in the intent-to-treat population with parasite, fever and gametocyte clearance, median clearance times and the proportion of patients with clearance at days 1, 2, and 3. Click here for file

Additional file 3 Key laboratory variables: baseline values, changes from baseline at days 3 and 7 and 28, and incidence of post-baseline grade 3 or 4 toxicity values for hepatic enzymes and total bilirubin. Click here for file
