# Supplementary material for: Pyronaridine-artesunate granules versus artemether-lumefantrine crushed tablets in children with Plasmodium falciparum malaria: a randomized controlled trial
Source: Malar J. 2012 Oct 31;11:364. doi: 10.1186/1475-2875-11-364 (PMC3566922; doi:10.1186/1475-2875-11-364)
Supplement: Additional file 1 — PCR-corrected day-28 adequate clinical and parasitological response rates in the per-protocol population by country and patient age. [file 1475-2875-11-364-S1.doc]

**Supplementary Data Table 1.** **PCR-corrected Day-28 Adequate Clinical and Parasitological Response Rates in the Per-Protocol Population by Country and Patient Age**

| Sub-group analysis | Pyronaridine-artesunate | Artemether-lumefantrine |
| --- | --- | --- |
| Country |  |  |
| Burkina Faso | 18/19 (94.7; 74.0–99.9) | 6/6 (100; 54.1–100) |
| DR Congo | 50/52 (96.2; 86.8–99.5) | 25/26 (96.2; 80.4–99.9) |
| Gabon | 52/52 (100; 93.2–100) | 27/27 (100; 87.2–100) |
| Ivory Coast | 64/69 (92.8; 83.9-97.6) | 30/31 (96.8; 83.3–99.9) |
| Kenya | 50/52 (96.2; 86.8–99.5) | 27/27 (100; 87.2–100) |
| Mali | 84/84 (100; 95.7–100) | 43/43 (100; 91.8–100) |
| Philippines | 11/11 (100; 71.5–100) | 7/7 (100; 59.0–100) |
| Age, years |  |  |
| <1 | 9/11 (81.8; 48.2–97.7) | 2/3 (66.7; 9.4–99.2) |
| 1–<5 | 135/141 (95.7; 91.0–98.4) | 61/61 (100; 94.1–100) |
| 5–12 | 185/187 (98.9; 96.2– 99.9) | 102/103 (99.0; 94.7–100) |

Data are n/N (%; 95% CI).
