# Supplementary material for: Pyronaridine-artesunate granules versus artemether-lumefantrine crushed tablets in children with Plasmodium falciparum malaria: a randomized controlled trial
Source: Malar J. 2012 Oct 31;11:364. doi: 10.1186/1475-2875-11-364 (PMC3566922; doi:10.1186/1475-2875-11-364)
Supplement: Additional file 2 — Number of patients in the intent-to-treat population with parasite, fever and gametocyte clearance, median clearance times and the proportion of patients with clearance at days 1, 2, and 3. [file 1475-2875-11-364-S2.doc]

**Additional file 2**

**Number of patients in the intent-to-treat population with parasite, fever and gametocyte clearance, median clearance times and the proportion of patients with clearance at days 1, 2, and 3**

| Outcome^a^ | Pyronaridine-artesunate | Artemether-lumefantrine |
| --- | --- | --- |
| Asexual parasite clearance, n/N (%)^b^ | 341/355 (96.1) | 175/180 (97.2) |
| Median clearance time, h (95% CI) | 24.1 (24.0–24.1) | 24.2 (24.1–32.0) |
| Patients with clearance, % (95% CI) at: |  |  |
| Day 1 | 49.3 (44.1–54.5) | 41.7 (34.5–48.9) |
| Day 2 | 94.6 (92.3–97.0) | 93.3 (89.7–97.0) |
| Day 3 | 96.1 (94.0–98.1) | 97.2 (94.8–99.6) |
| Fever clearance, n/N (%)^b^ | 274/278 (98.6) | 129/135 (95.6) |
| Median clearance time, h (95% CI) | 8.1 (8.0–8.1) | 8.1 (8.0–15.8) |
| Patients with clearance, % (95% CI) at: |  |  |
| Day 1 | 86.3 (82.3–90.4) | 79.3 (72.4–86.1) |
| Day 2 | 97.5 (95.6–99.3) | 94.1 (90.1–98.1) |
| Day 3 | 98.6 (97.2–100) | 95.6 (92.1–99.0) |
| Gametocyte clearance^b^ | 9/44 (20.5) | 7/26 (26.9) |
| Range of clearance time, h | 8.0–40.1 | 7.2–32.1 |
| Patients with clearance, % (95% CI) at: |  |  |
| Day 1 | 13.6 (3.5–23.8) | 23.1 (6.9–39.3) |
| Day 2 | 20.5 (8.5–32.4) | 26.9 (9.9–44.0) |
| Day 3 | 20.5 (8.5–32.4) | 26.9 (9.9–44.0) |

CI, confidence interval. Day 1 = 24 h after first dose; day 2 = 48 h after first dose; day 3 = 72 h after first dose.

^a^ Median clearance times, percentages of clearance at each and corresponding confidence intervals were calculated by Kaplan-Meier analysis. Patients without clearance by 72 h were censored at this time.

^b^ Observed values at end of therapy (day 3).
