# Supplementary material for: Pyronaridine-artesunate granules versus artemether-lumefantrine crushed tablets in children with Plasmodium falciparum malaria: a randomized controlled trial
Source: Malar J. 2012 Oct 31;11:364. doi: 10.1186/1475-2875-11-364 (PMC3566922; doi:10.1186/1475-2875-11-364)
Supplement: Additional file 3 — Key laboratory variables: baseline values, changes from baseline at days 3 and 7 and 28, and incidence of post-baseline grade 3 or 4 toxicity values for hepatic enzymes and total bilirubin. [file 1475-2875-11-364-S3.doc]

**Additional file 3**

**Key laboratory variables: baseline values, changes from baseline at days 3 and 7 and 28, and incidence of post-baseline grade 3 or 4 toxicity values for hepatic enzymes and total bilirubin**

| Parameter | Time/ toxicity | *n* | Pyronaridine-artesunate | *n* | Artemether-lumefantrine |
| --- | --- | --- | --- | --- | --- |
| Hemoglobin, g/dL | Baseline | 355 | 10.2 (1.3) [8.0, 14.2] | 180 | 10.3 (1.4) [8.0, 14.2] |
|  | Day 3 | 348 | –0.60 (1.2) [–4.2, 2.8] | 176 | –0.68 (1.2) [–5.4, 2.5] |
|  | Day 7 | 345 | –0.28 (1.1) [–3.2, 2.6] | 173 | –0.26 (1.1) [–3.0, 3.1] |
|  | Day 28 | 334 | 0.85 (1.3) [–2.8, 4.8] | 171 | 0.75 (1.4) [–3.8, 5.8] |
| Reticulocytes, % | Baseline | 114 | 2.0 (1.8) [0.3, 11.6] | 56 | 2.1 (1.6) [0.4, 6.7] |
|  | Day 3 | 110 | –0.1 (1.2) [–5.2, 3.0] | 53 | 0 (1.2) [–4.1, 3.3] |
|  | Day 7 | 107 | 0.6 (2.1) [–8.0, 8.2] | 50 | 0.5 (1.2) [–2.7, 4.9] |
|  | Day 28 | 85 | 0.3 (2.0) [–10.4, 3.8] | 42 | 0.3 (2.0) [–6.1, 4.7] |
| ALT, U/L | Baseline | 355 | 25.0 (12.1) [5, 105] | 180 | 25.8 (13.7) [5, 106] |
|  | Day 3 | 345 | 0.8 (16.8) [–47, 181] | 170 | 2.9 (57.5) [–32, 720] |
|  | Day 7 | 344 | 1.8 (37.5) [–46, 643] | 173 | –1.4 (22.8) [–57, 258] |
|  | Grade 3/4 | 355 | 1 (0.3) | 180 | 1 (0.6) |
| AST, U/L | Baseline | 355 | 43.5 (17.4) [7, 164] | 180 | 42.8 (15.6) [17, 116] |
|  | Day 3 | 346 | –0.7 (32.6) [–114, 459] | 171 | 0.2 (42.7) [–55, 477] |
|  | Day 7 | 342 | –2.0 (44.1) [–128, 735] | 173 | –4.2 (21.2) [–85, 163] |
|  | Grade 3/4 | 355 | 3 (0.8) | 180 | 1 (0.6) |
| ALP, U/L^a^ | Baseline | 347 | 195.7 (108.4) [5, 884] | 176 | 182.0 (95.5) [5, 622] |
|  | Day 3 | 336 | –6.3 (85.2) [–752, 428] | 166 | 9.5 (70.4) [–245, 324] |
|  | Day 7 | 336 | –5.6 (88.9) [–488, 330] | 169 | 12.6 (83.8) [–212, 450] |
| Total bilirubin, μmol/L | Baseline | 350 | 17.9 (12.5) [2.1, 128.5] | 177 | 18.4 (13.4) [0.5, 121.4] |
|  | Day 3 | 340 | –9.1 (11.8) [–122.2, 29.1] | 167 | –9.3 (15.4) [–114.6, 88.9] |
|  | Day 7 | 339 | –8.0 (12.1) [–119.5, 41.0] | 170 | –9.1 (12.9) [–107.7, 27.0] |
|  | Grade 3^b^ | 355 | 1 (0.3) | 180 | 2 (1.1) |
| Creatinine, μmol/L^a^ | Baseline | 349 | 41.8 (17.1) [7.8, 123.1] | 177 | 42.6 (18.0) [17.7, 106.1] |
|  | Day 3 | 339 | –3.5 (18.3) [–79.9, 44.2] | 167 | –2.9 (20.1) [–70.7, 108.0] |
|  | Day 7 | 339 | –3.4 (17.7) [–87.7, 44.2] | 170 | –3.6 (17.6) [–53.0, 62.8] |

Values are mean (SD) [minimum, maximum] except for grade 3/4 toxicity which is n (%).

Day-28 values were available for ALT, AST, ALP, total bilirubin and creatinine.

ALT, alanine aminotransferase; AST aspartate aminotransferase; ALP alkaline phosphatase.

^a^ There were no patients with grade 3/4 toxicity values for ALP or creatinine.

^b^ There were no patients with grade 4 toxicity values for total bilirubin.

ALT and AST grade 3 toxicity was 10–15 times the upper limit of normal and grade 4 toxicity was >15 times the upper limit of normal. Total bilirubin grade 3 toxicity was 3.0–7.5 times the upper limit of normal.
